# Seasonality of *Amblyomma americanum* (Acari: Ixodidae) Activity and Prevalence of Infection with Tick-Borne Disease Agents in North Central Oklahoma

**DOI:** 10.1089/vbz.2023.0009

**Published:** 2023-11-01

**Authors:** Kristin L. McClung, Kellee D. Sundstrom, Megan W. Lineberry, Amber N. Grant, Susan E. Little

**Affiliations:** ^1^ Department of Veterinary Pathobiology, College of Veterinary Medicine, Oklahoma State University, Stillwater, Oklahoma, USA.; ^2^ Veterinary Medicine Research and Development, Zoetis, Kalamazoo, Michigan, USA.

**Keywords:** *Amblyomma americanum*, *Borrelia*, *Ehrlichia*, phenology, seasonality, spotted fever group *Rickettsia*

## Abstract

**Background:**

*Amblyomma americanum* is the most common tick infesting both animals and humans in the southern United States and transmits a variety of zoonotic agents. The rise in tick-borne diseases (TBD) globally imparts a need for more active surveillance of tick populations to accurately quantify prevalence and risk of tick-borne infectious organisms. To better understand TBD risk in north central Oklahoma, this study aimed to describe the current seasonal activity of *A. americanum* in this region and investigate the seasonality of tick-borne infectious agents.

**Materials and Methods:**

Tick collections were performed twice a month for a duration of 2 years at a field site in Payne County, Oklahoma. Total nucleic acid was extracted from a subset of adult *A. americanum* and tested for *Rickettsia* spp., *Ehrlichia* spp., and *Borrelia* spp. using established PCR protocols.

**Results:**

Peak activity times for each life stage were observed, with adults primarily active 1 month earlier than historical seasonal trends describe, and male *A. americanum* active earlier in the year than female *A. americanum*. *Rickettsia* spp., *Ehrlichia chaffeensis*, *Ehrlichia ewingii*, and *Borrelia lonestari* were found in 26.4%, 6.1%, 2.5%, and 1.1% of adult *A. americanum*, respectively. No seasonal trend in spotted fever group *Rickettsia* spp. (SFGR) was observed in peak activity months.

**Conclusions:**

This study found an apparently shifting phenology for *A. americanum* adults in Oklahoma. While these results did not show a trend in SFGR, further investigation is needed to better understand the potential seasonality of infection prevalence within *A. americanum* across the expanding range of this vector, especially considering the extended activity of males in winter months.

## Introduction

Tick-borne diseases are increasing globally, with the lack of vaccines exacerbating the public health burden (Paules et al., 2018; Rosenberg et al., 2018; Saleh et al., 2021). Seasonality of tick-borne infections is usually tied to the phenology of the main tick vectors (Saleh et al., 2021). For example, peak activity of *Amblyomma americanum*, the lone star tick, coincides with most cases of human ehrlichiosis caused by *Ehrlichia chaffeensis* in the United States (US) (Demma et al., 2005; Mogg et al., 2020). However, the risk season for human ehrlichiosis can last as long as 7 months in Missouri and cases are reported in every month across the southern United States (Andrews et al., 2019: Mogg et al., 2020).

*A. americanum* is the most common tick collected from humans, domestic animals, wildlife, and habitat in the southern United States (Goddard, 2002; Kollars et al., 2000; Saleh et al., 2021; Saleh et al., 2019; Sundstrom et al., 2021). The broad feeding habits of *A. americanum* enhance its ability to bridge the gap between animal reservoirs and humans (Nicholson et al., 2019). High *A. americanum* populations are reliant on suitable forested and edge habitat for off-host survival of ticks as well as ample populations of white-tailed deer (*Odocoileus virginianus*), a preferred host for all three parasitic life stages, and ground-dwelling birds to provide additional hosts for immature stages (Bishopp and Trembley, 1945; Saleh et al., 2021).

Organisms transmitted by lone star ticks include *E. chaffeensis* and *Ehrlichia ewingii*, causative agents of ehrlichioses in humans and dogs; *Borrelia lonestari*, a putative agent of southern tick-associated rash illness and relapsing fever; and novel agents such as Heartland and Bourbon virus (Childs and Paddock, 2003; Dupuis et al., 2021; McClung and Little, 2023; Vazquez Guillamet et al., 2023).

In addition, *A. americanum* are commonly infected with *Rickettsia amblyommatis* and other spotted fever group *Rickettsia* spp. (SFGR), with prevalence estimates for SFGR from field-collected ticks ranging from 11.8% to as high as 95.6% depending on the region and life stage considered (Egizi et al., 2020; Heise et al., 2010; Mixson et al., 2006; Small and Brennan, 2021). Although more research is needed, *R. amblyommatis* has been implicated as a putative etiological agent of mild spotted fever rickettsiosis (SFR) in humans, which was previously misclassified as Rocky Mountain spotted fever (RMSF) (Apperson et al., 2008).

The rise in reports of mild SFR, serologic cross-reactivity between *Rickettsia* spp., and the apparently low prevalence of *Rickettsia rickettsii* in *Dermacentor variabilis*, the primary vector of the classic RMSF agent in North America, have led some to suggest that increasing SFR reports are instead due to infection with *R. amblyommatis* and other SFGR (Apperson et al., 2008; Delisle et al., 2016; Hecht et al., 2019; Openshaw et al., 2010; Stromdahl et al., 2011).

Historically, the peak activity for *A. americanum* in Oklahoma was August and September for larvae and nymphs, with adults peaking in late May and early June (Semtner and Hair, 1973). As changes in temperature and humidity continue, phenology and distribution of lone star ticks are expected to shift (McClung and Little, 2023; Raghavan et al., 2016; Springer et al., 2015). Previously, case reports of illnesses associated with *A. americanum* have paralleled peak activity times and known *A. americanum* distribution (Demma et al., 2005; Mogg et al., 2020). However, as the phenology of this vector continues to shift, active surveillance is needed to identify any corresponding potential shift in tick-borne disease risk (Colwell et al., 2011). In this article, structured tick collections and molecular assays were used to define the activity of *A. americanum* in north central Oklahoma and investigate the potential seasonality of infections they harbor.

## Materials and Methods

### Tick collection and identification

Ticks were collected using a combination of flags, drags, and dry ice traps, as previously described from September 2020 to August 2022 from a field site (5000-m^2^) in Payne County, Oklahoma (36.11052, −97.20596), consisting of ∼50% forest and 50% grassland habitat (Newman et al., 2019). At each tick collection, drags were used to sample half the grassland area (1250-m^2^) in 50-meter lengths for a total of 25 passes, and flags were used to sample understory in half the wooded area (1250-m^2^) in 50-meter lengths for a total of 25 passes. All adult and nymphal ticks collected from drags or flags were removed and placed in 70% ethanol. For larvae found on each pass, an estimate of number was made in increments of 50 and 15 representative larval ticks removed and placed in 70% ethanol; the remainder of larvae were removed from the cloth with adhesive tape before the next pass.

Dry ice traps (*n* = 16, each ∼0.1-m^2^) were each placed randomly in 16 quadrants in the other 2500-m^2^ of the site, with 8 traps placed in the forest habitat (1250-m^2^) and 8 traps in the grassland habitat (1250-m^2^) (Koch and McNew, 1981). Each trap was baited with ∼250 g of dry ice and allowed to sublimate for at least 90 min. All adult and nymphal ticks collected with dry ice traps were placed in 70% ethanol; larvae were enumerated, but not collected from tape. Personnel performing collections were provided disposable coveralls (Tyvek^®^; DuPont, Richmond, VA) as personal protective equipment (PPE) and regularly checked the outer layer for ticks; ticks found on PPE were collected with tape and recorded as “walking” collections (Chapman and Siegle, 2000).

Tick collections were repeated twice each month throughout the 24-month study period, weather permitting, for a total of 43 collections; 5 collection dates were missed due to inclement weather. Temperature, humidity, and wind speed were confirmed at the beginning and end of each collection for both habitat types with a handheld meter (Kestrel 3500 Delta T, Boothwyn, PA) and collections only performed on days without active precipitation when temperatures were above freezing and wind speeds were below 10 miles per hour. All collected ticks were identified to life stage, sex, and species based on morphology with the use of dichotomous keys (Keirans and Durden, 1998; Strickland et al., 1976) and stored at −20°C until tested for infectious agents.

### Infectious agent testing of *A. americanum*

A subset (*n* = 522) of adult field-collected *A. americanum* was selected for infectious organism testing. At least 100 adult *A. americanum* during each peak month were randomly selected for testing to assess infection seasonality with each month approximately balanced by the number of males and females. All adult *A. americanum* from nonpeak months were tested.

Each selected adult *A. americanum* was dorsoventrally dissected with sterile materials. Total nucleic acid was extracted from the internal contents using a commercial kit (DNeasy; Qiagen, Germantown, MD). Nested PCR was performed as previously described to detect gene fragments characteristic of *Rickettsia* spp. (17-kDa), *Ehrlichia* spp. (16S rRNA), and *Borrelia* spp. (flaB), and, when necessary, *Rickettsia* spp. identity confirmed with a second target (OmpA) (Dawson et al., 1996; Heise et al., 2010; McBride et al., 1996; Moore et al., 2003; Murphy et al., 1998; Sumner et al., 2007). All PCR was performed in designated laboratory areas with site-specific reagents.

Positive controls were included to confirm amplification, and negative controls were included at each step from extraction through amplification to ensure contamination would be detected. Amplicons were purified with a commercial kit (Wizard^®^ SV Gel and PCR Clean-Up System; Promega, Madison, WI) and sequenced directly with an ABI 3730 capillary sequencer (Applied Biosystems, Foster City, CA) at the Oklahoma State University Molecular Core Facility (Stillwater, OK). Upon verification of high-quality electropherograms by visual inspection and routine editing, sequences were compared to all available sequences published in GenBank (https://blast.ncbi.nlm.nih.gov/Blast.cgi) and a curated set of sequences for *Rickettsia* spp. (CP003334; EF689727; GU723477), *Ehrlichia* spp. (NR_074500; NR_044747), and *Borrelia* spp. (AY166716; CP001205).

### Statistical analyses

The sample size was determined in R (version 3.5.2) with a proportion power calculation for binomial distribution (*α* = 0.05, power = 0.80) based on estimated infectious organism prevalence, as previously published, including 33.6% for SFGR, 3.3% for *E. chaffeensis*, 5.4% for *E. ewingii*, and 2.9% for *B. lonestari* (Bacon et al., 2005; Mixson et al., 2006; Small and Brennan, 2021; Steiert and Gilfoy, 2002). Peak activity times were defined as months (inclusive) when 90% or more of each life stage was present. QuickCalcs-GraphPad (www.graphpad.com) was used for all proportions and corresponding confidence intervals (95% CI).

Collection method comparisons for each life stage were performed with a single-factor ANOVA and Tukey Honest Significance Difference (HSD) Test in Excel with a Q statistic critical value of 3.67. Habitat comparisons (wooded vs. grassland) for each life stage were performed with a two-sample *t*-test in Excel. A chi-square test was used to compare monthly *Rickettsia* spp. prevalence in adult ticks across peak months. Fischer's exact tests were used to compare male and female activity and *Rickettsia* spp. prevalence. All statistics were performed using a significance level of *α* = 0.05.

## Results

### Tick collection

A total of 7990 *A. americanum* were collected over the 24 months of the study, including 941 adults (463 males and 478 females), 2767 nymphs, and 4282 larvae. A majority of *A. americanum* (4328/7990; 54.2%) were collected on dry ice traps and the remainder were collected with flags (*n* = 2969; 37.2%) and drags (*n* = 404; 5.1%). A few *A. americanum*, including 61 adults, 128 nymphs, and 100 larvae, were collected through walking (*n* = 289; 3.6%); area of collection (wooded vs. grassland) was not recorded for walking ticks. Dry ice traps collected 849 adults, 2529 nymphs, and 950 larvae; flags collected 24 adults, 93 nymphs, and 2852 larvae; and dragging collected 7 adults, 17 nymphs, and 380 larvae.

**FIG. 1.**
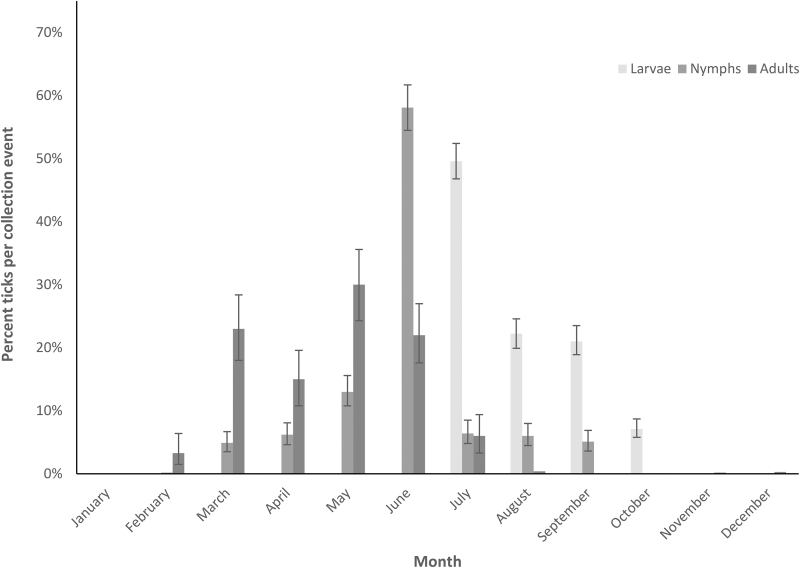
Seasonality of *Amblyomma americanum* throughout the year.

*A. americanum* larvae were collected on flags significantly more than on dry ice traps or drags, or by walking (Tukey's HSD *Q* = 3.18–4.60). Nymphs and adults of *A. americanum* were both collected through dry ice traps significantly more than on flags or drags, or by walking (Tukey's HSD *Q* = 5.46–8.72). All stages of *A. americanum* were more commonly collected in wooded areas (6501/7701; 84.4%) compared to grasslands (1200/7701; 15.6%), including adults (*p* = 0.004), nymphs (*p* = 0.031), and larvae (*p* = 0.027). Adult *A. americanum* were primarily collected March–June (847/941; 90.0%); nymphs March–August (2613/2767; 94.4%); and larvae July–September (3933/4282; 91.9%) (Fig. 1).

**FIG. 2.**
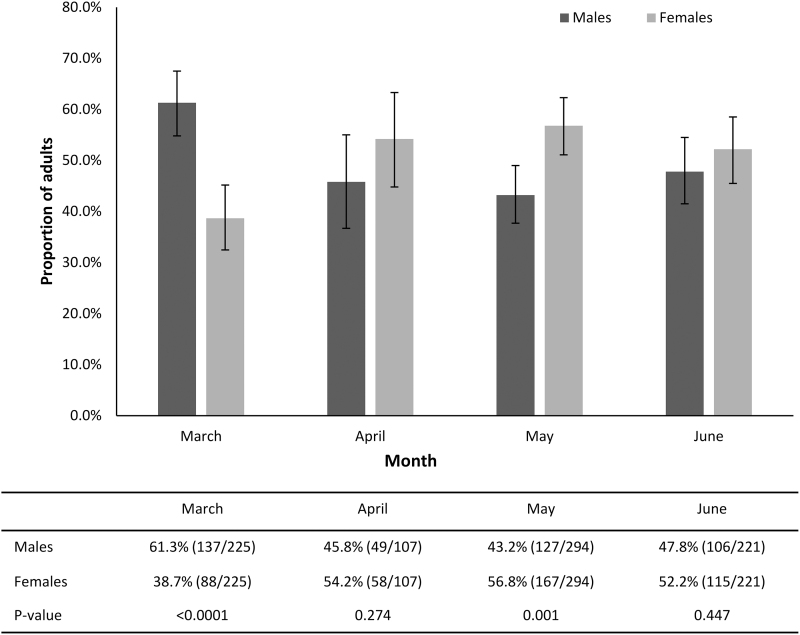
Proportion of male and female *A. americanum* during peak months of activity (March through June).

Male *A. americanum* were collected more than females in March (*p* < 0.0001) and female *A. americanum* were collected more than males in May (*p* = 0.001) (Fig. 2). Other ticks collected included 31 *Ixodes scapularis* adults (18 males and 13 females) collected on flags (*n* = 19), drags (*n* = 3), and dry ice traps (*n* = 9), primarily from October to January (28/31; 90.3%); most (24/31; 77.4%) *I. scapularis* were collected from wooded areas. One *Amblyomma maculatum* female was collected by walking in July.

### Prevalence and seasonality of infectious agents in *A. americanum*

Infectious agents were detected in 33.7% (176/522; 95% CI 29.8–37.9) of adult *A. americanum* (Table 1), including 34.9% (152/435; 95% CI 30.6–39.5) collected during peak months of activity (March–June), and 27.6% (24/87; 95% CI 19.3–37.8) collected during other times of the year. SFGR were detected in 26.4% (138/522; 95% CI 22.8–30.4) of adult *A. americanum*; sequencing revealed most (89.9%) (124/138; 95% CI 83.6–94.0) were 100% identical to that reported from *R. amblyommatis* (CP003334) although sequence of an unclassified *Rickettsia* sp. previously described from *A. americanum* in Texas (EF689727) was identified in 10.1% of ticks (14/138; 95% CI 6.0–16.4).

**Table 1.** Overall Prevalence of *Rickettsia* spp., *Ehrlichia* spp., and *Borrelia lonestari* in Adult *Amblyomma americanum*

Infectious agents	Prevalence	95% CI
All agents	33.7% (176/522)	29.8–37.9
*Rickettsia* spp.	26.4% (138/522)	22.8–30.4
*Rickettsia amblyommatis*	23.8% (124/522)	20.3–27.6
Uncultured *Rickettsia* sp.	2.7% (14/522)	1.6–4.5
*Ehrlichia* spp.	8.6% (45/522)	6.5–11.4
*Ehrlichia chaffeensis*	6.1% (32/522)	4.4–8.6
*Ehrlichia ewingii*	2.5% (13/522)	1.4–4.3
*Borrelia* spp.	1.1% (6/522)	0.5–2.6
*Borrelia lonestari*	1.1% (6/522)	0.5–2.6

CI, confidence interval.

*E. chaffeensis* and *E. ewingii* were identified in 6.1% (32/522; 95% CI 4.4–8.6) and 2.5% (13/522; 95% CI 1.4–4.3) of ticks, respectively; all sequences were identical to those previously described (NR_074500; NR_044747). *B. lonestari* was detected in 1.1% (6/522; 95% CI 0.5–2.6) of adult *A. americanum*; sequence was identical to that previously reported (AY166716) (Table 1). Coinfections were identified in 13 ticks, including 5 ticks with *R. amblyommatis* and *E. chaffeensis*; four ticks with *R. amblyommatis* and *E. ewingii*; two ticks with *E. chaffeensis* and *E. ewingii*; one tick with *R. amblyommatis* and *B. lonestari*; and one tick with *Rickettsia* sp. and *E. chaffeensis.*

SFGR prevalence was not significantly different in male (25.4%; 64/252; 95% CI 20.4–31.1) and female ticks (27.5%; 74/269; 95% CI 22.5–33.1) (*χ*^2^ = 0.298, df = 1, *p* = 0.585), or in ticks collected in different months of peak activity (*χ*^2^ = 2.215, df = 3, *p* = 0.529) (Table 2). Cochran-Armitage trend analysis did not reveal seasonal patterns in the prevalence of SFG *Rickettsia* spp. infection (*p* = 0.1057). Sample size in this study was not adequate to evaluate distribution or trends for agents occurring at lower prevalence (*i.e.*, *Ehrlichia* spp., *B. lonestari*).

**Table 2.** Monthly Prevalence of Spotted Fever Group *Rickettsia* spp. in Adult *Amblyomma americanum* Collected During Peak Activity (March Through June)

Month	Rickettsia spp. Prevalence	95% CI
March	20.8% (25/120)	14.5–29.0
April	30.7% (31/101)	22.5–40.3
May	24.1% (26/108)	16.9–33.0
June	33.0% (35/106)	24.8–42.5

An additional 87 adult *A. americanum* tested were collected at off-peak times and were not included in seasonal analysis for prevalence of spotted fever group *Rickettsia* spp.

## Discussion

*A. americanum* adults in this study were primarily active 1 month earlier in the year compared to historic seasonality reported in Oklahoma (Semtner and Hair, 1973). In the southeastern United States, *A. americanum* adult peak activity time has been reported to occur anywhere from April to June (Gleim et al., 2014). Climate models have predicted a shift in phenology for *A. americanum* in the southern United States, including earlier activity times and enhanced winter survival (Briske et al., 2015; Ludwig et al., 2016). Other recent studies have described *A. americanum* nymphs and adults questing much earlier than previously reported (Noden et al., 2022a; Raghavan et al., 2021).

In addition, in this study, male *A. americanum* predominated earlier in the year, including in March when peak adult questing was first noted, and female *A. americanum* were more commonly identified in May (Fig. 2). Other recent studies describing *A. americanum* have also reported significantly more males than females active early in the year (Raghavan et al., 2021). This difference may be due to the greater ability of male lone star ticks to survive cold temperatures relative to females (Needham et al., 1996). Examination of cattle and white-tailed deer in late fall and winter also reveals a preponderance of male *A. americanum* (Barnard, 1981).

The prevalence of SFGR observed in this study is consistent with those previously reported for *A. americanum* from the region, which ranges from 11.8% to 37.3% (Mixson et al., 2006; Noden et al., 2022b; Small and Brennan, 2021). No difference was evident in *Rickettsia* spp. prevalence between male and female ticks, an observation that has been reported previously and highlights risk of infection from both sexes of *A. americanum* (Mixson et al., 2006).

Because male *A. americanum* must feed to become sexually mature and survive on hosts, the prolonged winter activity of males represents a heightened risk of lone star tick-borne infections outside the months typically considered to be peak risk. This study confirmed SFGR in *A. americanum* during nonpeak months, although the sample size limits confidence of prevalence estimates. Most reports of SFR occur during peak tick activity time, but reports are documented in every month of the year (Center for Disease Control and Prevention, 2022). The findings in this article highlight a risk of transmission from the bite of *A. americanum* year round.

Fitness costs to ticks from infection with pathogens have been described in several tick-pathogen systems, such as *R. rickettsii*-infected *Dermacentor andersoni* exhibiting increased mortality, while molting (Niebylski et al., 1999); *Borrelia burgdorferi*-infected *I. scapularis* questing at lower heights and being less active (Lefcort and Durden, 1996); and *B. burgdorferi*-infected *Ixodes persulcatus* moving more slowly (Romashchenko et al., 2012).

Similarly, a recent report documented that *A. americanum* ticks infected with *R. amblyommatis* spend significantly less time questing than noninfected ticks (Richardson et al., 2022). If SFGR infection confers a fitness cost to *A. americanum*, a lower prevalence of infection may be found in ticks recovered later in the year. Alternatively, if infection affords a fitness advantage, as seen in *I. scapularis* infected with *Anaplasma phagocytophilum* exhibiting increased winter survival (Neelakanta et al., 2010), an increase in infection prevalence may be evident later in the year. Although no seasonal trend of *Rickettsia* spp. prevalence was identified in this study, prevalence may vary over time and should be considered in future studies.

*Ehrlichia* spp. prevalence in adult *A. americanum* ranges from 1% to 17.3% and *B. lonestari* prevalence ranges from 0% to 2.9%; this study found similar results, as well as a 2.5% coinfection rate in ticks, which is comparable to other reports (Mixson et al., 2006; Small and Brennan, 2021; Steiert and Gilfoy, 2002; Varela et al., 2004). Coinfections in ticks may reduce acquisition of *Rickettsia parkeri*, shifting prevalence (Wright et al., 2015).

Coinfections are also common in vertebrates. For example, 19.6% of dogs in the southern United States with antibodies to *Ehrlichia* spp. had been infected with both *E. ewingii* and *E. chaffeensis* (Beall et al., 2012), and coinfections with *Ehrlichia* spp. and *Rickettsia* spp. are commonly documented by PCR in dogs and wild canids from the region (Barrett et al., 2014; Starkey et al., 2014; Starkey et al., 2013). Coinfections with tick-borne agents are associated with the development of more severe disease and have important implications for human and veterinary medicine (Little, 2010; Rocha et al., 2022).

Limitations of the study include focus on one tick species, few ticks collected in nonpeak months, and predominance of *R. amblyommatis*. Inclement weather prevented tick collection in 2 of the 24 months of the study, reducing total number of *A. americanum* available for testing and thus constraining overall confidence in trend analysis of seasonality of SFG *Rickettsia* spp. Nonetheless, large numbers of *A. americanum* were collected and the infection prevalence estimates were consistent with those previously reported (Mixson et al., 2006; Small and Brennan, 2021). This study focused on common SFG *Rickettsia* spp., but alternate assays may have revealed other agents (Lydy et al., 2020). Despite these limitations, this study found infection prevalence estimates consistent with previous reports and infections were still identified during off-peak times.

## Conclusions

This study highlights an apparently shifting phenology of *A. americanum* in the southcentral United States, with different male and female activity peaks. Lone star ticks carry infectious agents all year, posing a year-round risk, especially in winter when males are still active. Further monitoring of tick activity and infection is necessary due to the importance of *A. americanum* for human and animal health, especially in the southern United States.
